# Hydrometrocolpos in Infants: Etiologies and Clinical Presentations

**DOI:** 10.3390/children9020219

**Published:** 2022-02-07

**Authors:** Mi-Chi Chen, Yao-Lung Chang, Hsun-Chin Chao

**Affiliations:** 1 Division of Pediatric Gastroenterology, Department of Pediatrics, Chang Gung Children’s Medical Center, Chang Gung Memorial Hospital, Taoyuan City 33305, Taiwan; mp1250@cgmh.org.tw; 2 Department of Obstetrics and Gynecology, Chang Gung Memorial Hospital, Taoyuan City 33305, Taiwan; j12054@cgmh.org.tw; 3 College of Medicine, Chang Gung University, Taoyuan City 33302, Taiwan

**Keywords:** hydrometrocolpos, imperforate hymen, persistent urogenital sinus, cloacal malformation, Herlyn-Werner-Wunderlich syndrome

## Abstract

Hydrometrocolpos (HMC) is a rare condition where fluids or secretions accumulate in the vagina (hydrocolpos) or up to the uterus (hydrometrocolpos). This case series study reports three infants with different etiologies and presentations of HMC and aims to review literature for proper workup upon initial diagnosis. The first neonate antenatally presented with a huge cystic mass. HMC secondary to imperforate hymen was proved, and hymenotomy was performed at 2 days of age. The second participant presented with persistent urogenital sinus and hematopoietic chimerism, possibly due to transfusion from her twin brother via placenta anastomoses. At 2 months of corrected age, she had difficult defecating, and sonogram revealed HMC with normal appearance of uterus and ovaries. Regular follow-ups and surgical reconstruction will be conducted before puberty. The third patient had cloacal malformation and multiple congenital anomalies at birth. Vesicovaginal fistula-related HMC was detected and managed with surgical drainage in the neonate stage. The girl began menstruation with dysmenorrhea at 12 years. The image studies demonstrated hematometrocolpos secondary to left-side hemivaginal septum, uterine didelphy, and ipsilateral renal agenesis, indicating Herlyn–Werner–Wunderlich syndrome. HMC can be diagnosed easily via sonogram. Careful external genitalia examinations help to identify persistent urogenital sinus or cloacal malformation. Occasionally, the HMC may be part of syndrome manifestations or associated with sex chromosome anomalies. Clinicians may conduct surveillance of renal, cardiac, and skeletal systems as well as chromosome study for early diagnosis and management.

## 1. Introduction

Hemato/hydrometrocolpos is a rare condition caused by distal vaginal obstruction with accumulation of blood or mucus secretions in the vagina and uterus. The reported incidence of hydrometrocolpos is around 0.006% [1]. Hemato/hydrometrocolpos is caused by congenital urogenital anomalies or acquired etiologies such as infection, trauma, or sexual abuse [2,3,4,5]. The most common congenital cause is imperforate hymen [1], and the other associated structural anomalies include transverse vaginal septum, distal vaginal agenesis, persistent urogenital sinus, and cloacal malformation [6,7]. Hydrometrocolpos (HMC) can present as an antenatal pelvic cystic mass with or without hydroureteronephrosis under sonogram examinations. After birth, HMC may manifest as a palpable intraabdominal mass lesion or a vulvoperineal mass, and may combine with urogenital sinus or cloacal malformation. Vaginal retention of menstrual blood at puberty, which is hematometrocolpos, results in painful primary amenorrhea and pelvic mass [8]. The mass may also cause compression effects, such as hydroureteronephrosis, urinary retention, and constipation [3].

We herein report three infants of congenital HMC with different anatomical causes and clinical presentations.

## 2. Case Series

### 2.1. Case 1

**Figure 1:**
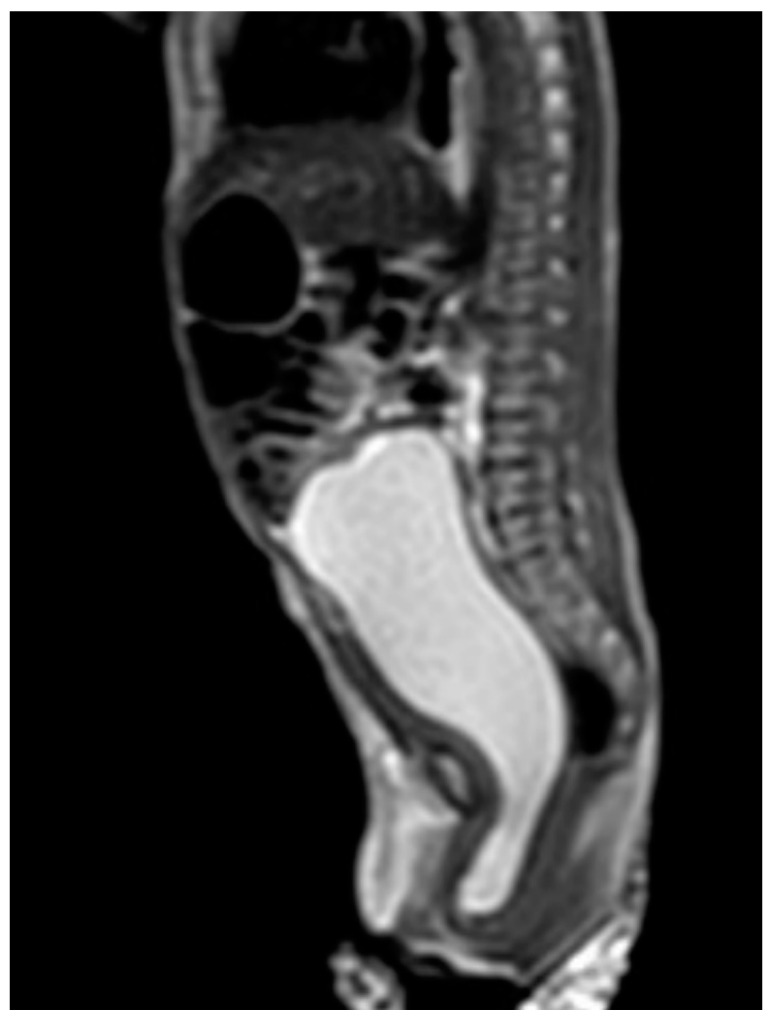
MRI of case 1: hydrometrocolpos manifested as an intraabdominal huge cystic mass.

Case 1 was born at 36 weeks of gestation. An 8 cm cystic mass in the lower abdomen was detected on antenatal ultrasound from 32 weeks of gestation. The patient presented with a bulging mass over the vulva area. Magnetic resonance imaging (MRI) (Figure 1) showed a 9.1 cm cystic mass in the pelvic cavity, which extended to the vaginal orifice and caused left hydroureter, suggesting an imperforated hymen. Hymenotomy with a cross excision was performed, and 50 mL of milky fluids was drained. The patient is now 4 years, 4 months old, healthy without other medical issues.

### 2.2. Case 2

**Figure 2:**
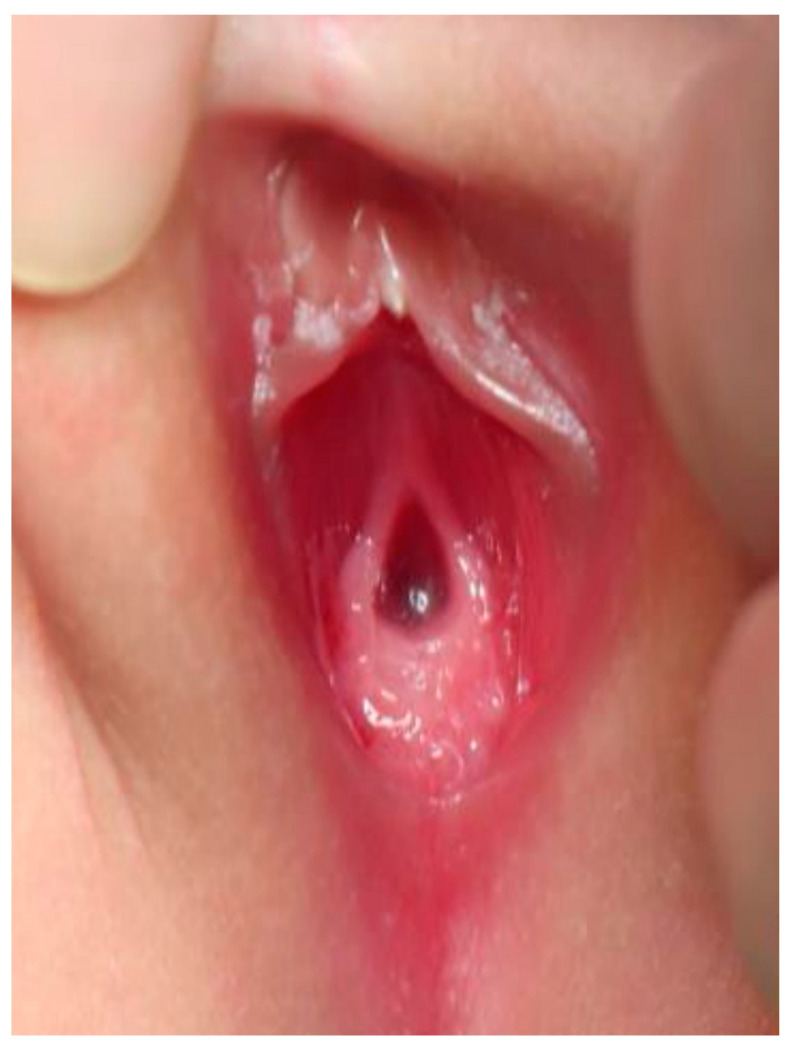
External genitalia of case 2: single orifice of urogenital sinus.

Case 2 was a twin A, conceived with in vitro fertilization (IVF), born at 29 weeks of gestation. Antenatal ultrasound showed that the female fetus was a donor and the male fetus was a recipient of twin–twin transfusion syndrome (TTTS) at 20 weeks of gestation. Laser therapy was performed at 22 weeks of gestation. Cordocentesis of twin A revealed the karyotype of 46, XY, which conflicted with the female genitalia appearance and a donor of TTTS. Thus, the chromosome study of peripheral lymphocytes was rechecked and reported 46, XY (82%)/46, XX (18%). The sex-determining region of the Y-chromosome (SRY) gene was positive. The external genitalia presented with normal anus and a single genital opening, suspecting a common sheath of urogenital sinus (Figure 2).

**Figure 3:**
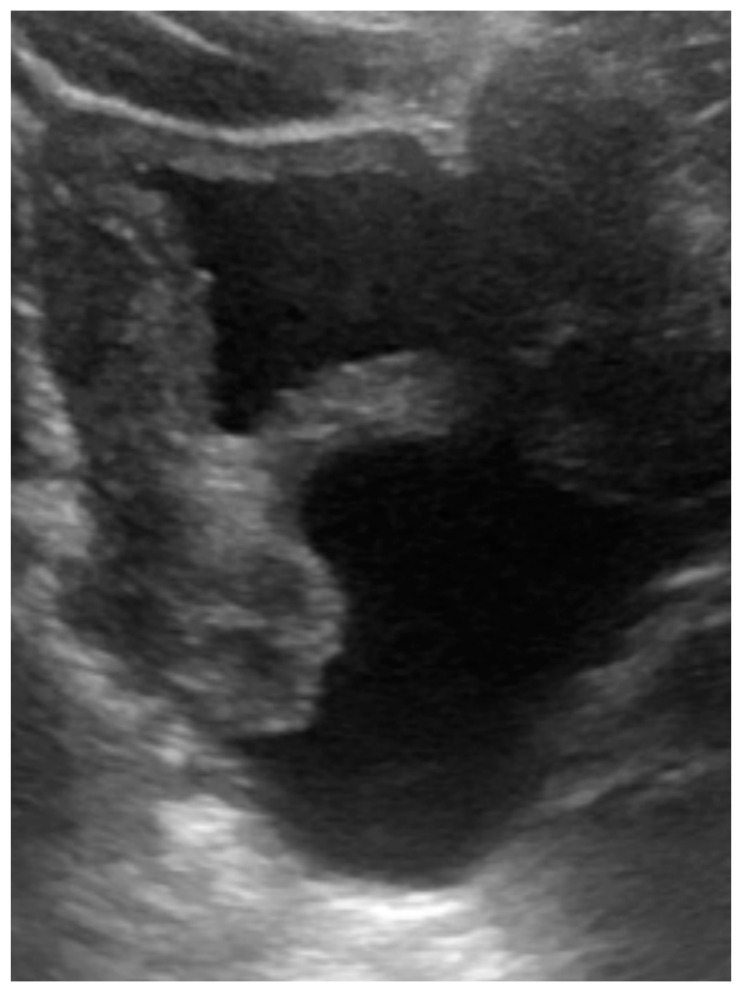
Ultrasound of case 2: hydrocolpos.

The patient presented with defecation difficulty at 2 months of corrected age. The ultrasound (Figure 3) and MRI demonstrated hydrocolpos, and presence of uterus and bilateral ovaries. Cystoscopy failed to identify the opening of the vagina. Currently, the girl is 8 months of corrected age with fair development and growth. Follow-up ultrasound still revealed hydrocolpos, but without compression effects. MRI examination will be arranged before puberty for further surgical correction.

### 2.3. Case 3

**Figure 4:**
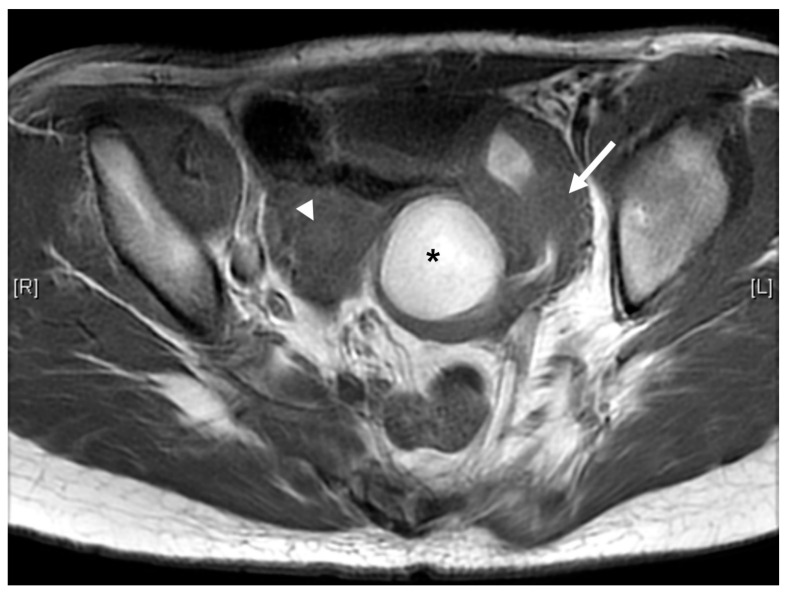
MRI of case 3: hematometrocolpos (asterisk) and uterine didelphy (arrow and arrowhead).

**Figure 5:**
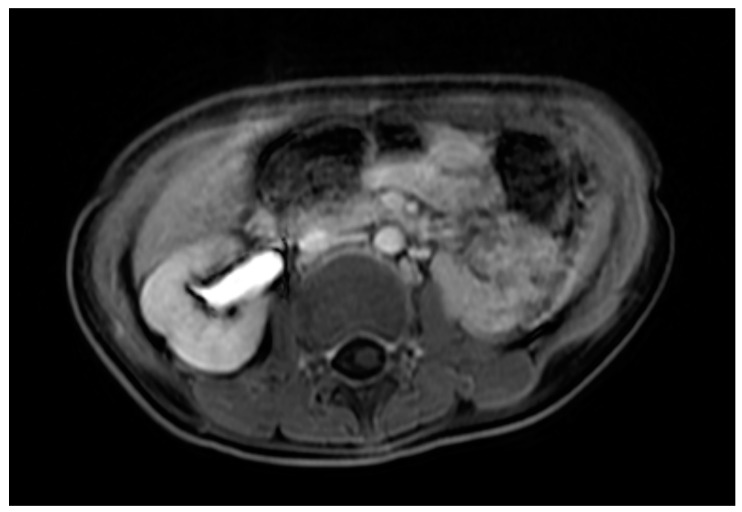
MRI of case 3: left renal agenesis and right hydroureter.

Case 3 was a female infant born at 34 weeks of gestation. The antenatal ultrasound showed fetal ureteral obstruction and oligohydramnios. She had a poorly developed vestibule with a small orifice near the clitoris and high-type imperforate anus, indicating cloacal malformation. A double-barrel colostomy and drainage of 30 mL turbid urine of the orifice of the common channel, possibly from the distended vagina, was performed at 5 hours of age. Abdominal ultrasound at 1 day of age showed a 7.5 cm cystic mass in the lower abdomen, but it decreased in size on the next day. Water instillation via urinary catheter visualized the urinary bladder and enlarged the cystic mass on real-time ultrasound, suggesting HMC with vesicovaginal fistula. The patient received spinal lipoma excision at 8 months of age for the lipomyelomeningocele, and repair for tetralogy of Fallot at 1 year of age. Posterior sagittal anorectovaginourethroplasty using the Pena method was performed at 1 year, 8 months of age, and the distal vagina was replaced by a 5 cm length of rectum. She began menstruating at 12 years, 10 months, which was associated with cyclic abdominal pain, and the MRI detected uterine didelphys with left hematometrocolpos, hemivagina, and left renal agenesis (Figure 4 and Figure 5). Hysteroscopy was performed via the neovagina, and one bulging mass in the left side was observed. The right uterus cavity was normal with smooth endometrium. Herlyn–Werner–Wunderlich syndrome was diagnosed. The patient anticipates left hemi-hysterectomy and transcervical resection of the vaginal septum.

## 3. Discussion

The three infants in this study had different clinical presentations and were diagnosed at different times (Table 1). The structural anomalies associated with HMC may involve only the genital tract (Müllerian duct abnormalities) or they may combine with urinary tract (urogenital sinus) and anorectal tract (cloacal malformation) anomalies. Simplified classification of HMC [9] was conducted on the basis of obstruction level and severity of malformation. Five types were classified: type I (imperforate hymen), type II (vaginal septum), type III (distal vaginal atresia), type IV (vaginal atresia with persistent urogenital sinus), and type V (vaginal atresia with cloacal anomaly). External genitalia inspection may provide clues for initial classification. In the neonates and young infants, the HMC accumulates with two types of fluids [10]: mucus, secreted by the glands of the uterus and cervix, and urine, secondary to urogenital or cloacal malformations.

Presentations of HMC depend on the degree of compression of the surrounding structures. Frequently, the urinary tract is compressed causing varying degrees of hydronephrosis as in case 1 and 3, as previous reports mentioned [11,12,13]; pressure on the bowel to cause constipation, as in case 2, is not common [8,14]. In more severe compression effects, the infants may present with acute urinary retention and intestinal obstruction [15,16,17]. HMC can sometimes be part of several syndromes. One case series [12] enrolled 20 participants prenatally diagnosed with HMC, and postnatal follow-up showed four patients with McKusick Kaufman syndrome or Bardet–Biedl syndrome, three patients with Mayer–Rokitansky–Küster–Hauser syndrome, and one patient with Herlyn–Werner–Wunderlich syndrome. Patients with congenital HMC should be searched for other anomalies such as postaxial polydactyly, congenital heart disease of McKusick Kaufman syndrome/Bardet–Biedl syndrome [18,19], and renal, skeletal, ear, and cardiac malformations of Mayer–Rokitansky–Küster–Hauser syndrome [20,21]. It is difficult to distinguish McKusick Kaufman syndrome and Bardet–Biedl syndrome in the neonatal period. Re-evaluation in childhood for learning difficulties, obesity, retinitis pigmentosa, and renal abnormalities may lead to the diagnosis of Bardet–Biedl syndrome. Typical manifestations of these syndromes are summarized in Table 2. These features, especially complex congenital heart diseases, influence outcomes of patients with HMC. Our case 3 presented with the triad of uterine didelphy, hemivaginal septum, and ipsilateral renal agenesis, which were typical findings of Herlyn–Werner–Wunderlich syndrome. The patients with Herlyn–Werner–Wunderlich syndrome are usually asymptomatic until menarche, which presents with cyclical dysmenorrhea and a lower abdominal mass [22,23,24]. Interestingly, case 3 was diagnosed with cloacal malformation and Herlyn–Werner–Wunderlich syndrome, which lead to hydrometrocolpos in the neonate stage and hematometrocolpos in the adolescent stage, respectively. In our search, the co-existence of both diseases was not be reported.

We suggest a peripheral blood chromosome study for patients with HMC for disorders of sexual development and a sex chromosome anomaly survey. HMC had been reported associated with 47, XXX, 45, X/46, XX mosaicism and 45, X [25,26]. Generally, the reproductive outcomes of normal karyotypes are good. All of our three cases had a peripheral blood chromosome study. Case 2 had hematopoietic chimerism and positive SRY gene; cases 1 and 3 confirmed with 46, XX. A similar condition has been reported in dizygotic twins (male and female) conceived by IVF [27]. The 8-year-old girl clinically presented with clitoromegaly. Hematopoietic chimerism: 46, XX (53%) and 46, XY (47%) with presence of SRY gene was confirmed. However, the karyotype of gonads reported 46, XX without SRY gene. Hematopoietic chimerism in twins may occur from the transfer of hematopoietic stem cells through a common placenta [28]. Our case 2 and her twin brother had TTTS from 20 weeks of gestation, although the female infant was a donor. The mixing of hematopoietic stem cells through the vascular anastomoses in placenta was possible. Individuals with hematopoietic chimerism may have normal sexual development. Case 2 had normal appearance of uterus and ovaries under image studies. A case report [29] showed a fertile female presented with 46, XY karyotype of peripheral lymphocyte but 46, XX of fibroblasts of ovarian and muscular tissues and skin. Further observation for fertility function is required after the surgical correction of the urogenital sinus.

Surgical intervention of HMC varied from surgical drainage to correction of genital malformations according to the complexity of anomaly [9]. Huge HMC, as in case 1, requires urgent drainage to prevent HMC rupture. Case 3 had more complicated surgical corrections because of cloacal and multiple congenital anomalies. Twenty-eight percent of cloacal patients have associated HMC, and it is generally suggested to drain the HMC to avoid complications such as recurrent urinary tract infections, pyocolpos, sepsis, failure to thrive and ruptured HMC [30]. Generally, the outcomes of simple HMC related to imperforate hymen and normal karyotype are good. However, patients with complex cloacal malformation or Müllerian anomalies often require multiple reconstruction surgeries. Mental health problems including anxiety and depression, infertility, and pain management for long-term pelvic pain, pain associated with menstruation, urination, and intercourse, are important issues requiring multidisciplinary team care [31,32].

## 4. Conclusions

Hydrometrocolpos in infants is a rare condition. The presentations include an intraabdominal cystic mass, which may be evident in utero, and mass-related compression effects such as hydroureteronephrosis, urinary retention, constipation, and bowel obstruction. Associated structural anomalies in the urinary tract (persisted urogenital sinus) and anorectal tract (cloacal malformation) should be clarified for surgical planning. In addition, the hydrometrocolpos is sometimes part of syndromic manifestations. Careful evaluation of skeletal anomalies (postaxial polydactyl; fused vertebrae), congenital heart diseases, and renal anomalies (unilateral agenesis; ectopic) and chromosome studies are important for early diagnosis and proper management.

## Figures and Tables

**Table 1:** Summary of clinical findings in our three cases.

	Case 1	Case 2	Case 3
Gestational age	36 weeks	29 weeks, IVF, Twin A	34 weeks
Chromosome study	46, XX	46, XY (82%)/46, XX (18%)	46, XX
Other anomalies	Nil	Hematopoietic chimerism	Tetralogy of Fallot Lipomyelomeningocele Left renal agenesis
Age of HMC presentation	Antenatal (GA 32 weeks)	2 months of corrected age	At birth Adolescent
HMC classification	Type I (Imperforate hymen)	Type IV (Persistent urogenital sinus)	Type V (Cloacal malformation) Type II (Vaginal septum, relate to HHW syndrome)
HMC presentation	Abdominal cystic mass Hydroureter	Constipation	Aabdominal cystic mass; Dysmenorrhea
HMC management	Hymenotomy	No drainage, future surgical reconstruction	Cyst drainage Surgical reconstruction

Abbreviations: HMC: hydrometrocolpos, IVF: in vitro fertilization, GA: gestational age, HHW syndrome: Herlyn–Werner–Wunderlich syndrome.

**Table 2:** Syndromes associated with hydrometrocolpos.

	Typical Manifestations
McKusick Kaufman syndrome [18]	Hydrometrocolpos (95% of females)
	Postaxial polydactyly (98%)
	Congenital heart diseases (14%)
Bardet–Biedl syndrome [18,19]	Primary features: Genital anomalies (59–98%): Hydrometrocolpos (81% of females) Postaxial polydactyly (63–81%) Rod–cone dystrophy (93%) Obesity (72–92%) Learning difficulties (61%) Renal abnormalities (53%)
	Secondary features: speech delay, developmental delay, diabetes mellitus, dental anomalies, congenital heart diseases, brachydactylyl/syndactyly, ataxia/poor coordination, anosmia/hyposmia
Mayer–Rokitansky–Küster–Hauser syndrome [20,21]	Type I: isolated Congenital aplasia of the uterus and the upper part of the vagina Normal development of secondary sexual characteristics Normal 46, XX karyotype
	Type II: syndromic Renal (unilateral agenesis, ectopia of kidneys or horseshoe kidney) Skeletal (Klippel–Feil anomaly: fused vertebrae, mainly cervical) Hearing defects Rare: cardiac and digital anomalies (syndactyly, polydactyly)
Herlyn–Werner–Wunderlich syndrome [22,23]	Didelphys uterus
	Obstructed hemivagina
	Ipsilateral renal agenesis

## References

[1] Vitale V., Cigliano B., Vallone G. (2013). Imperforate hymen causing congenital hydrometrocolpos. J. Ultrasound.

[2] Berkowitz C.D., Elvik S.L., Logan M. (1987). A simulated "acquired" imperforate hymen following the genital trauma of sexual abuse. Clin. Pediatr..

[3] Tanitame K., Tanitame N., Urayama S., Ohtsu K. (2021). Congenital anomalies causing hemato/hydrocolpos: Imaging findings, treatments, and outcomes. Jpn. J. Radiol..

[4] Hwang H.J., Lim H.W., Han Y.S., Choi J.I., Kim M.J. (2016). Hematocolpos as a Result of Delayed Treatment of Acute Straddle Injury in an Adolescent Girl. Case Rep. Obstet. Gynecol..

[5] Scott S.M., Schlaff W. (2004). Hematocolpos associated with a remote history of chronic vaginitis and a diagnostic vaginal biopsy: A case report. J. Pediatr. Adolesc. Gynecol..

[6] Cerrah Celayir A., Kurt G., Sahin C., Cici I. (2013). Spectrum of etiologies causing hydrometrocolpos. J. Neonatal. Surg..

[7] Winkler N.S., Kennedy A.M., Woodward P.J. (2012). Cloacal malformation: Embryology, anatomy, and prenatal imaging features. J. Ultrasound Med..

[8] Lui C., Chan T., Fung H., Tang S. (2010). A Retrospective Study on Imperforate Hymen and Haematometrocolpos in a Regional Hospital. Hong Kong J. Emerg. Med..

[9] Khanna K., Sharma S., Gupta D.K. (2018). Hydrometrocolpos etiology and management: Past beckons the present. Pediatr. Surg. Int..

[10] Sharma D., Murki S., Pratap O.T., Irfan G., Kolar G. (2015). A case of hydrometrocolpos and polydactyly. Clin. Med. Insights Pediatr..

[11] Aggarwal S., Kumar A. (2003). Fetal hydrocolpos leading to Pierre Robin sequence: An unreported effect of oligohydramnios sequence. J. Perinatol..

[12] Mallmann M.R., Reutter H., Mack-Detlefsen B., Gottschalk I., Geipel A., Berg C., Boemers T.M., Gembruch U. (2019). Prenatal Diagnosis of Hydro(metro)colpos: A Series of 20 Cases. Fetal Diagn. Ther..

[13] Silva Í.S., Martello R., Mendes A., Chaves A. (2021). Urinary Retention Due to Hematocolpos. Acta Med. Port..

[14] Heckmann R., de la Fuente F.A., Heiner J.D. (2015). Pediatric urinary retention and constipation: Vaginal agenesis with hematometrocolpos. West. J. Emerg. Med..

[15] Arriola-Montenegro L., Arriola-Montenegro J., Pia-Balmaceda M., Celis-Albujar C., Riva-Moscoso A., Cabanillas-Lozada P., Velásquez-Huarcaya V. (2021). Hydrometrocolpos and Post-axial Polydactyly Complicated With Acute Intestinal Obstruction and Hydroureteronephrosis. Cureus.

[16] Hatti R.B., Badakali A.V., Vanaki R.N., Samalad M.S. (2013). Mckusick-kaufman syndrome presenting as acute intestinal obstruction. J. Neonatal. Surg..

[17] Okoro P.E., Obiorah C., Enyindah C.E. (2016). Experience with neonatal hydrometrocolpos in the Niger Delta area of Nigeria: Upsurge or increased recognition?. Afr. J. Paediatr. Surg..

[18] Slavotinek A.M., Biesecker L.G. (2000). Phenotypic overlap of McKusick-Kaufman syndrome with bardet-biedl syndrome: A literature review. Am. J. Med. Genet..

[19] Forsythe E., Kenny J., Bacchelli C., Beales P.L. (2018). Managing Bardet-Biedl Syndrome-Now and in the Future. Front Pediatr..

[20] Herlin M.K., Petersen M.B., Brännström M. (2020). Mayer-Rokitansky-Küster-Hauser (MRKH) syndrome: A comprehensive update. Orphanet J. Rare Dis..

[21] Morcel K., Camborieux L., Guerrier D. (2007). Mayer-Rokitansky-Küster-Hauser (MRKH) syndrome. Orphanet J. Rare Dis..

[22] Del Vescovo R., Battisti S., Di Paola V., Piccolo C.L., Cazzato R.L., Sansoni I., Grasso R.F., Zobel B.B. (2012). Herlyn-Werner-Wunderlich syndrome: MRI findings, radiological guide (two cases and literature review), and differential diagnosis. BMC Med. Imaging.

[23] Vo Nhu Q., Le Trong B., Nguyen Thanh T. (2021). Herlyn-Werner-Wunderlich syndrome: A report of three cases in adolescents and adult woman. Radiol. Case Rep..

[24] Zhang H., Ning G., Fu C., Bao L., Guo Y. (2020). Herlyn-Werner-Wunderlich syndrome: Diverse presentations and diagnosis on MRI. Clin. Radiol..

[25] Koeberl D.D., McGillivray B., Sybert V.P. (1995). Prenatal diagnosis of 45,X/46,XX mosaicism and 45,X: Implications for postnatal outcome. Am. J. Hum. Genet..

[26] Hood O.J., Hartwell E.A., Shattuck K.E., Rosenberg H.S. (1990). Multiple congenital anomalies associated with a 47,XXX chromosome constitution. Am. J. Med. Genet..

[27] Martos-Moreno G., Campos C., Flores R., Yturriaga R., Pérez-Jurado L.A., Argente J. (2013). Blood cell chimerism in dizygotic twins conceived by in vitro fertilization. An. Pediatr..

[28] Jang J.H., Jung H., Kim J.H., Park W.S., Kim S.H. (2010). Blood chimerism in a dizygotic dichorionic pregnancy. Korean J. Lab. Med..

[29] Sudik R., Jakubiczka S., Nawroth F., Gilberg E., Wieacker P.F. (2001). Chimerism in a fertile woman with 46,XY karyotype and female phenotype. Hum. Reprod..

[30] Bischoff A., Levitt M.A., Breech L., Louden E., Peña A. (2010). Hydrocolpos in cloacal malformations. J. Pediatr. Surg..

[31] Schall K., Parks M., Nemivant S., Hernandez J., Weidler E.M. (2019). Pelvic pain in patients with complex mullerian anomalies including Mayer-Rokitansky-Kuster-Hauser syndrome (MRKH), obstructed hemi-vagina ipsilateral renal anomaly (OHVIRA), and complex cloaca. Semin. Pediatr. Surg..

[32] Acién P., Acién M. (2016). The presentation and management of complex female genital malformations. Hum. Reprod. Update.

